# Supplementary material for: RAB20 Promotes Proliferation via G2/M Phase through the Chk1/cdc25c/cdc2-cyclinB1 Pathway in Penile Squamous Cell Carcinoma
Source: Cancers (Basel). 2022 Feb 22;14(5):1106. doi: 10.3390/cancers14051106 (PMC8909501; doi:10.3390/cancers14051106)
Supplement: Supplementary file 1 [file cancers-14-01106-s001.zip › cancers-1519289-supplementary.pdf]

cancers

Supplementary Materials

RAB20 Promotes Proliferation via G2/M Phase through the Chk1/cdc25c/cdc2-cyclinB1 Pathway in Penile Squamous Cell Carcinoma

Xingliang Tan [1,2,3,†], Gangjun Yuan [4,5,†], Yanjun Wang [1,2,3], Yuantao Zou [1,2,3], Sihao Luo [1,2,3], Hui Han [1,2,3], Zike Qin [1,2,3], Zhuowei Liu [1,2,3], Fangjian Zhou [1,2,3], Yanling Liu [2,3,6,*] and Kai Yao [1,2,3,*]

1 Department of Urology, Sun Yat-sen University Cancer Center, Guangzhou 510060, China; tanxl1@sysucc.org.cn (X.T.); wangyj@sysucc.org.cn (Y.W.); zouyt1@sysucc.org.cn (Y.Z.); luosh@sysucc.org.cn (S.L.); hanhui@sysucc.org.cn (H.H.); qinzk@sysucc.org.cn (Z.Q.); liuzhw@sysucc.org.cn (Z.L.); zhoufj@sysucc.org.cn (F.Z.)
2 State Key Laboratory of Oncology in Southern China, Guangzhou 510060, China
3 Collaborative Innovation Center of Cancer Medicine, Guangzhou 510060, China
4 Department of Urology Oncological Surgery, Chongqing University Cancer Hospital, Chongqing 400030, China; yuangj@cqu.edu.cn
5 Chongqing Key Laboratory of Translational Research for Cancer Metastasis and Individualized Treatment, Chongqing University Cancer Hospital, Chongqing 400030, China
6 Department of Operating Room, Sun Yat-sen University Cancer Center, Guangzhou 510060, China
* Correspondence: liuyl@sysucc.org.cn (Y.L.); yaokai@sysucc.org.cn (K.Y.)
† Xingliang Tan and Gangjun Yuan contributed equally to this study.

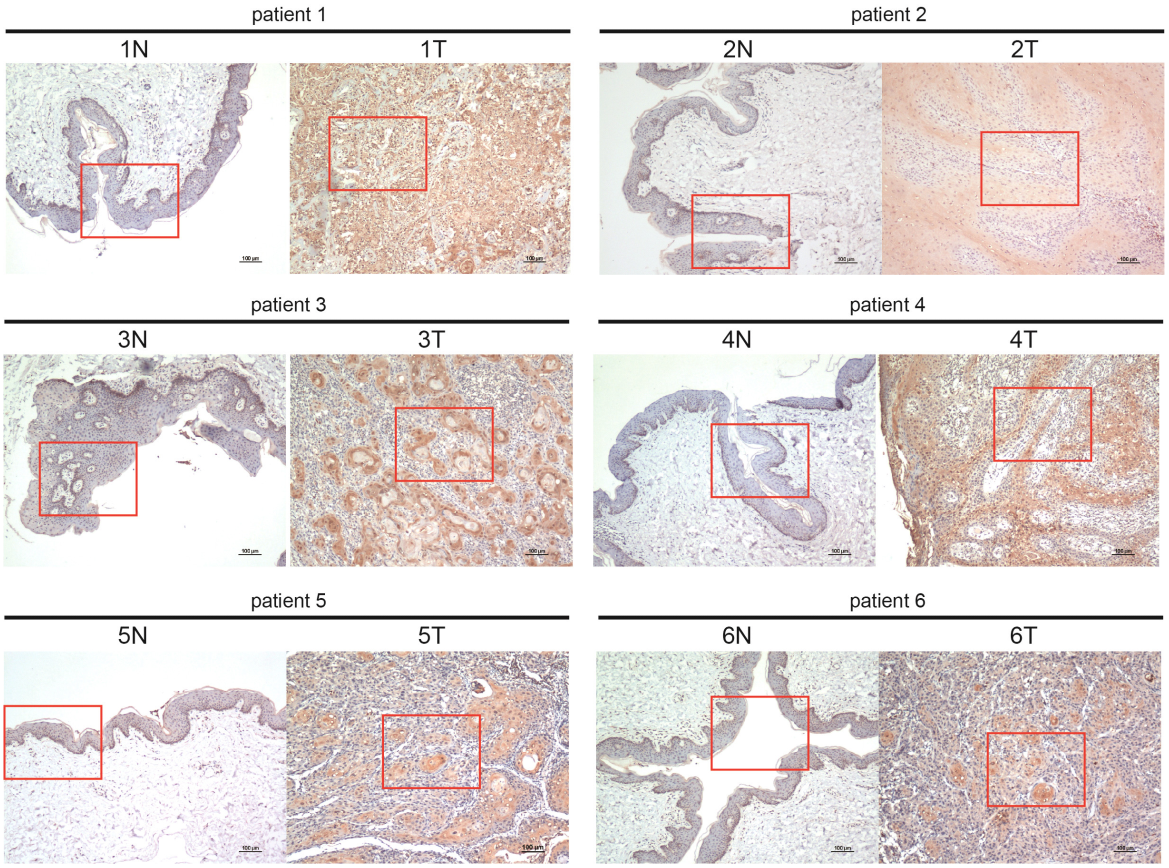

Figure S1. Immunohistochemical staining of RAB20 in 6 pairs of penile carcinoma and corresponding normal tissues. RAB20 protein was overexpressed in 6 pairs of penile tumor tissues comparing with normal squamous epithelium tissues (magnification: 100X). The red square area was enlarged and the images were shown in Figure 1E. N, normal tissues; T, penile carcinoma tissues.

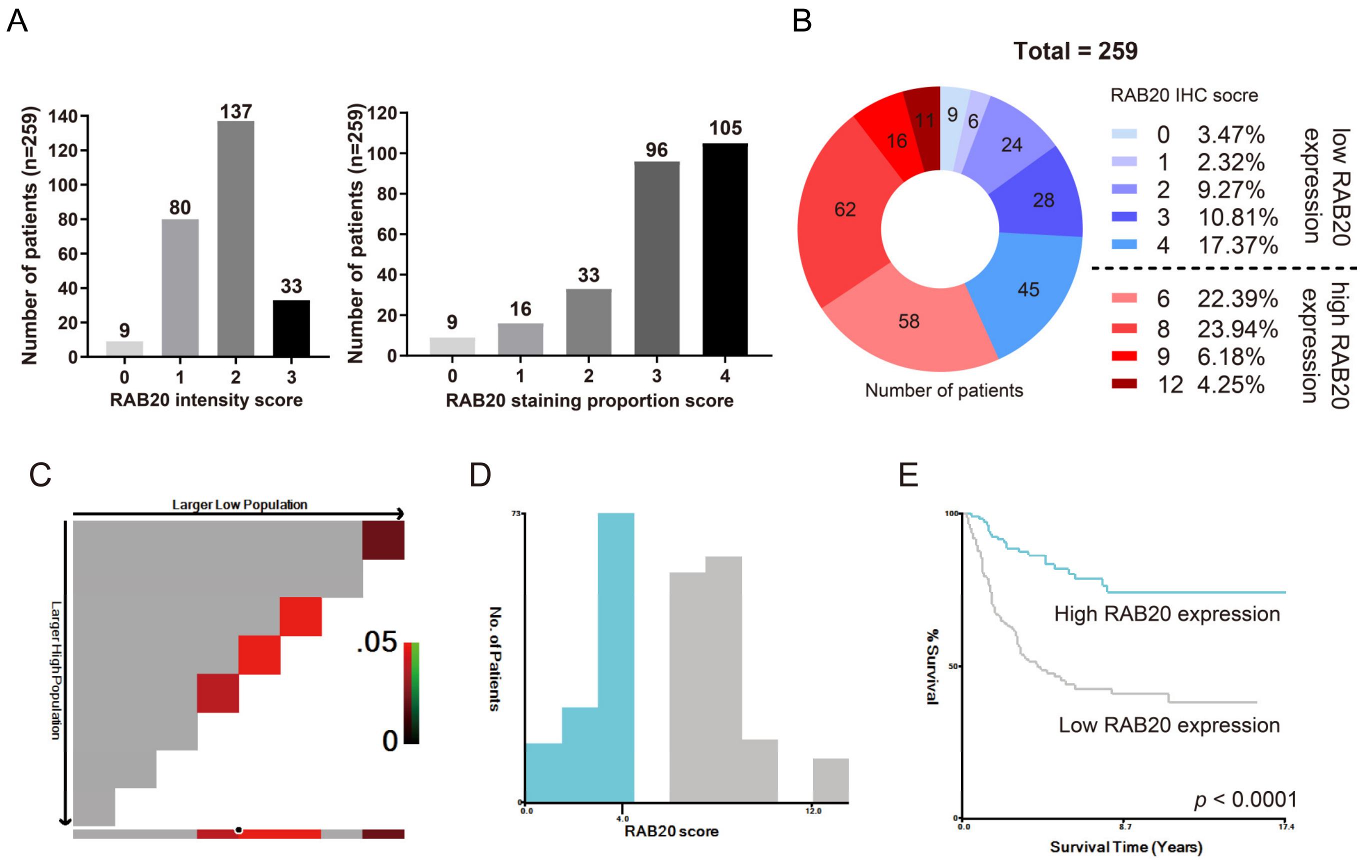

Figure S2. The cutoff value of RAB20 was calculated by X-tile software. (**A**) The IHC score of RAB20 staining scores were multiplied by the intensity score (0 for no staining, 1 for weak, 2 for clear and 3 for strong) and the staining area score (1 for 1-10%, 2 for 10-40%, 3 for 40%-70% and 4 for 70% above). The frequency of RAB20 intensity score and staning proportion score are shown. (**B**) The pie graph showed that the number and proportion of patients with different RAB20 IHC scores. (**C**) The diagram displayed the χ^2 log-rank values when the patients were divided into two groups. The optimal cutoff value (H score= 4, 0-4 points were low RAB20 expression and 6-12 points were high RAB20 expression) is highlighted by the black spot on the lower axis, (**D**) and the distribution is displayed in a histogram of the cohort by the X-tile software. (**E**) Kaplan–Meier survival analysis indicated that high RAB20 expression is associated with poor survival outcomes. IHC, immunohistochemistry; PSCC, penile squamous cell carcinoma.

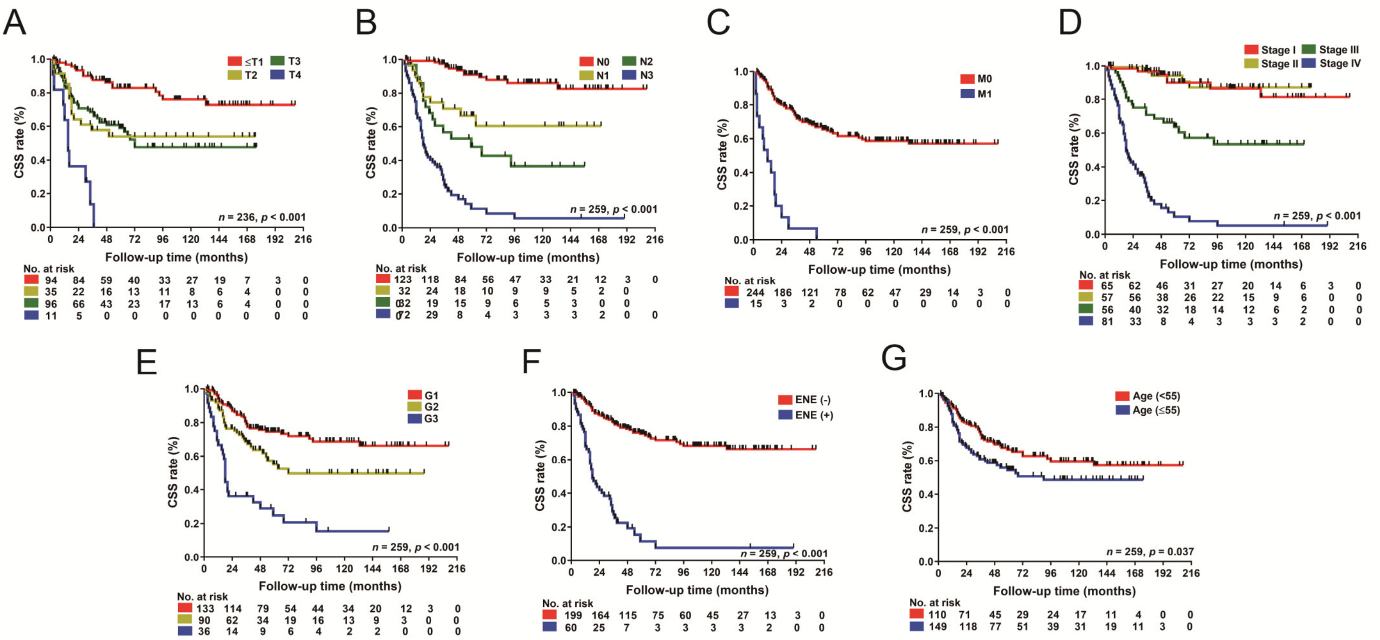

Figure S3. Relationship between clinical and pathological features and survival in 259 PSCC patients. Kaplan–Meier survival curves indicated that (**A**) T grade, (**B**) N grade, (**C**) metastasis, (**D**) clinical stage, (**E**) pathological grade, (**F**) extranodal extension, and (**G**) age were associated with CSS in 259 PSCC patients. The clinical and pathological features were determined based on the TNM Staging System for Penile Cancer (8th ed., 2017). PSCC, penile squamous cell carcinoma.

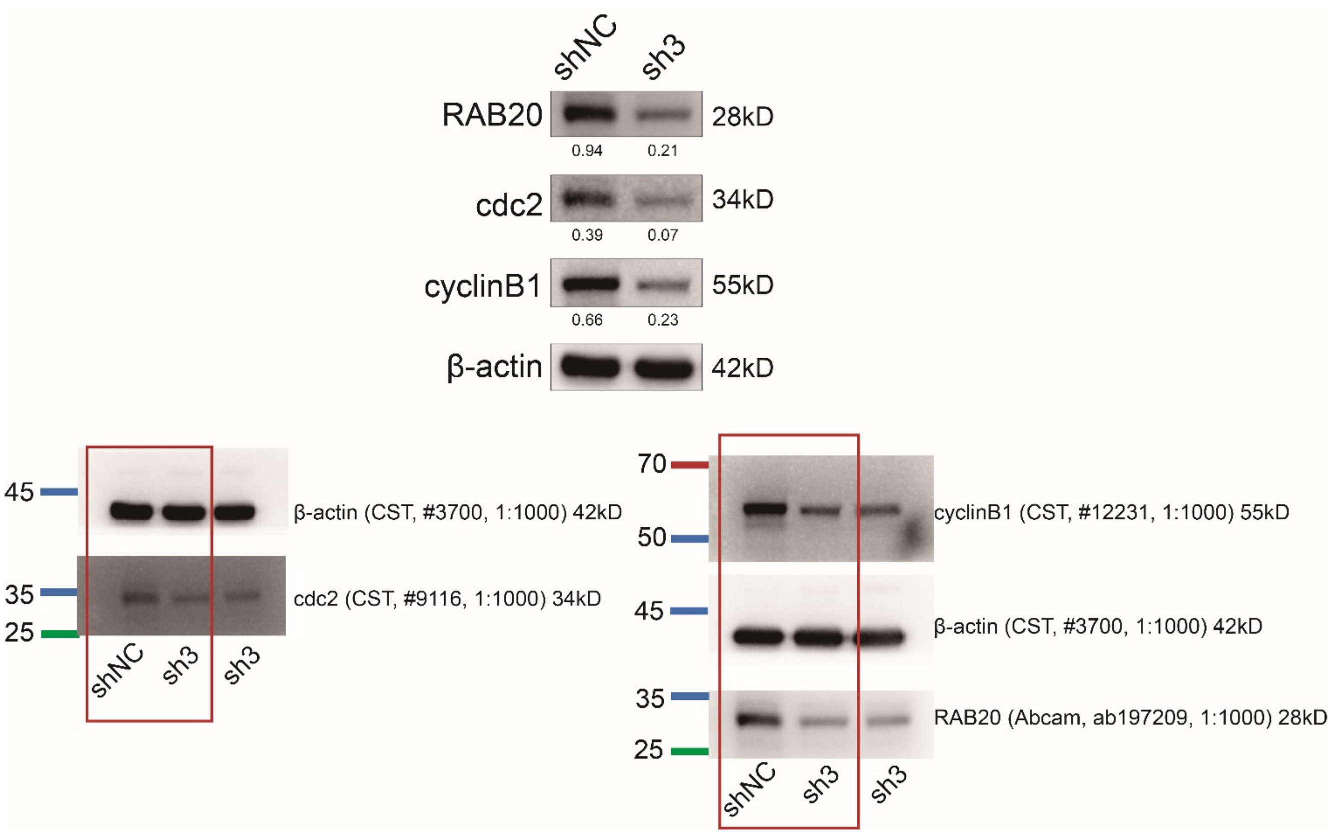

Figure S4. The protein expression of RAB20 and downsteam targets in tumors of the xenografted nude mice. Subcutaneous tumors of BALB/c nude mice in the RAB20-sh3 and the shNC groups were harvested. Western bolt confirmed that the protein expression of RAB20 was decreased in RAB20-sh3 group and its downstream targets cdc2 and cyclinB1 were also inhibited.

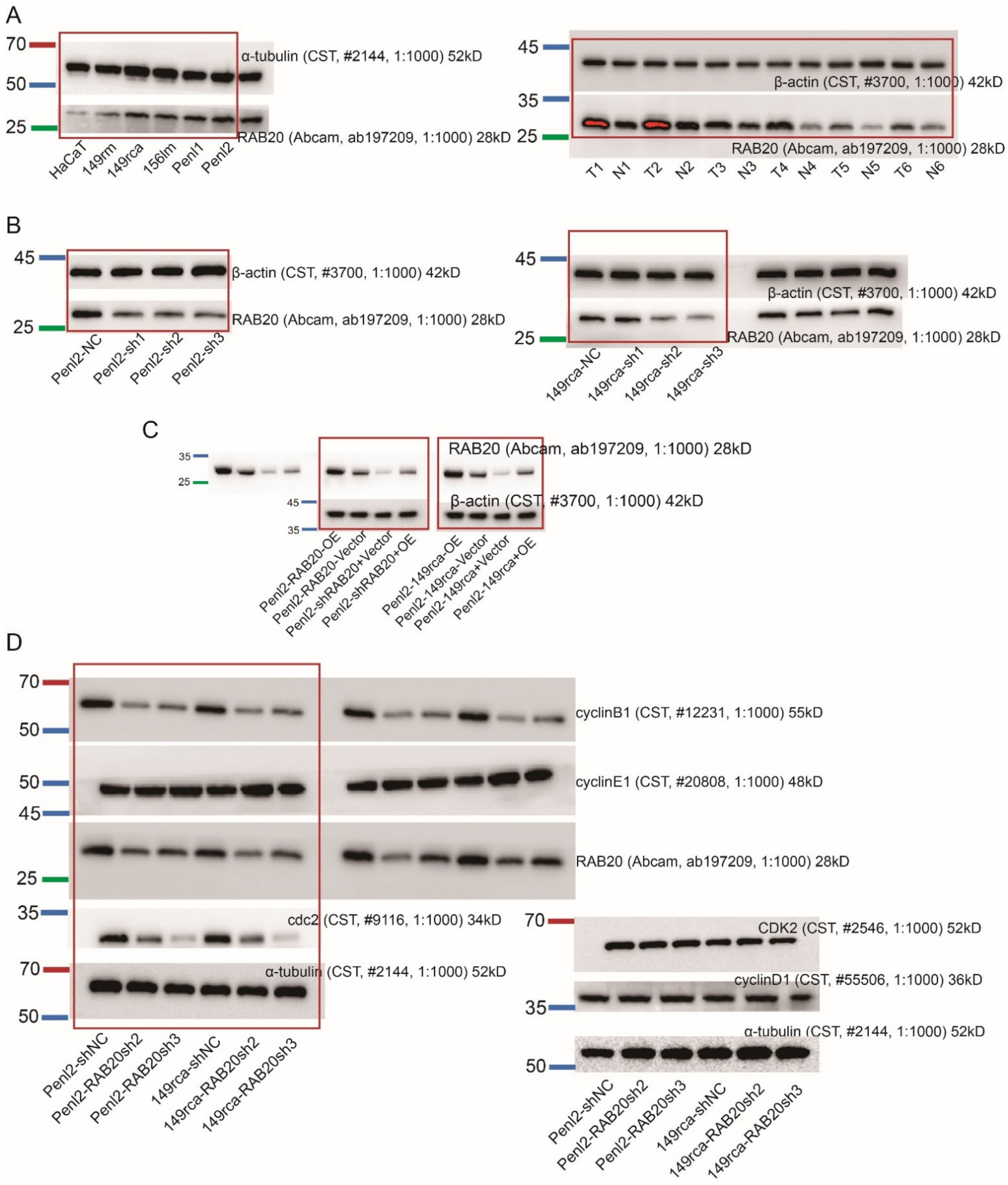
A
70
50
25
α-tubulin (CST, #2144, 1:1000) 52kD
RAB20 (Abcam, ab197209, 1:1000) 28kD
HaCaT
149rm
149rca
156lm
Penl1
Penl2
45
35
25
β-actin (CST, #3700, 1:1000) 42kD
RAB20 (Abcam, ab197209, 1:1000) 28kD
T1
N1
T2
N2
T3
N3
T4
N4
T5
N5
T6
N6
B
45
25
β-actin (CST, #3700, 1:1000) 42kD
RAB20 (Abcam, ab197209, 1:1000) 28kD
Penl2-NC
Penl2-sh1
Penl2-sh2
Penl2-sh3
45
25
β-actin (CST, #3700, 1:1000) 42kD
RAB20 (Abcam, ab197209, 1:1000) 28kD
149rca-NC
149rca-sh1
149rca-sh2
149rca-sh3
C
35
25
45
35
RAB20 (Abcam, ab197209, 1:1000) 28kD
β-actin (CST, #3700, 1:1000) 42kD
Penl2-RAB20-OE
Penl2-RAB20-Vector
Penl2-shRAB20+Vector
Penl2-shRAB20+OE
Penl2-149rca-OE
Penl2-149rca-Vector
Penl2-149rca+Vector
Penl2-149rca+OE
D
70
50
50
45
25
35
70
50
cyclinB1 (CST, #12231, 1:1000) 55kD
cyclinE1 (CST, #20808, 1:1000) 48kD
RAB20 (Abcam, ab197209, 1:1000) 28kD
cdc2 (CST, #9116, 1:1000) 34kD
α-tubulin (CST, #2144, 1:1000) 52kD
Penl2-shNC
Penl2-RAB20sh2
Penl2-RAB20sh3
149rca-shNC
149rca-RAB20sh2
149rca-RAB20sh3
70
35
50
CDK2 (CST, #2546, 1:1000) 52kD
cyclinD1 (CST, #55506, 1:1000) 36kD
α-tubulin (CST, #2144, 1:1000) 52kD
Penl2-shNC
Penl2-RAB20sh2
Penl2-RAB20sh3
149rca-shNC
149rca-RAB20sh2
149rca-RAB20sh3

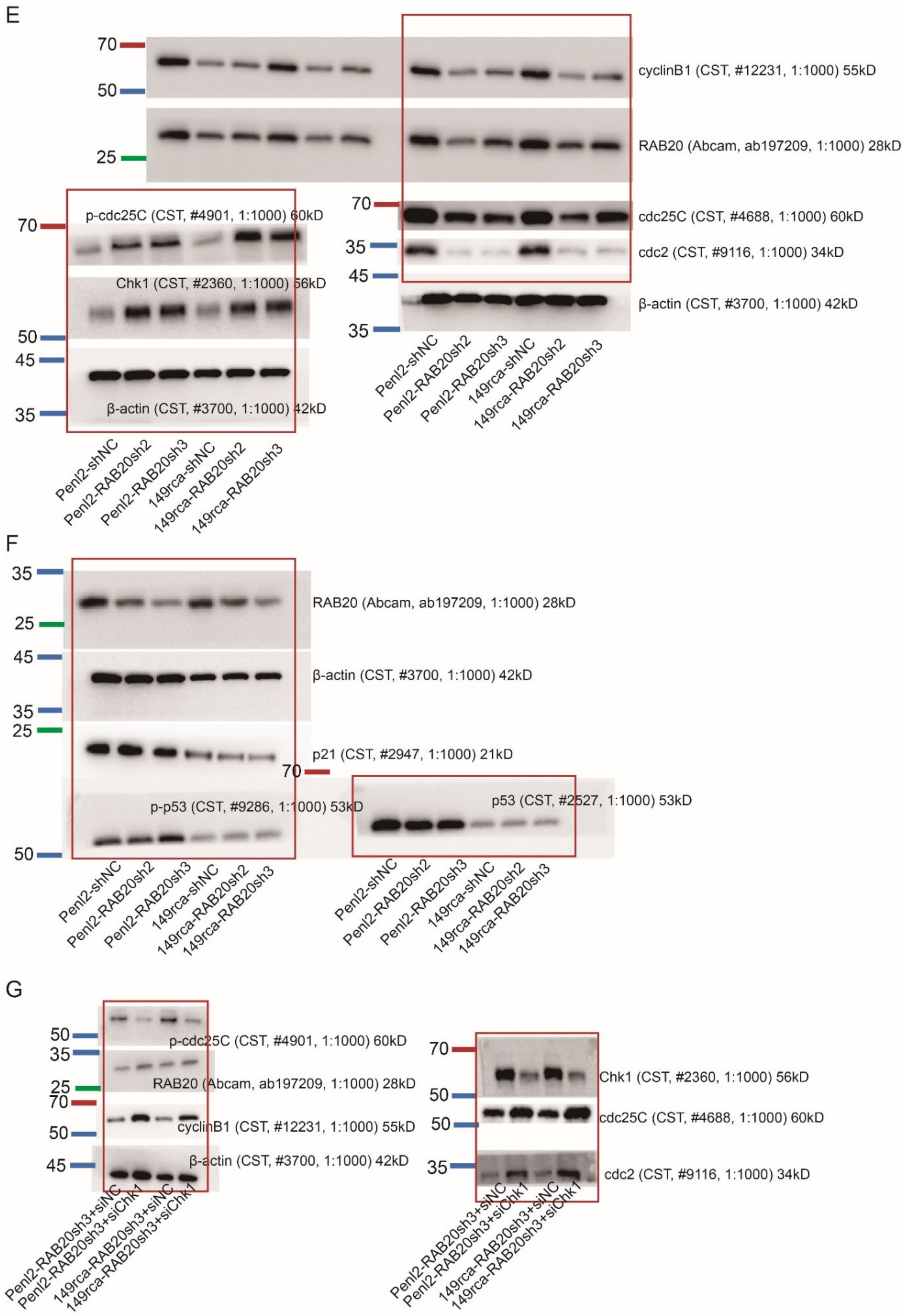

Figure S5. Original uncropped Western blots of Figure 1C and D, Figure 3A, Figure 4A, Figure 5 B, C, D, F.

Table S1. The baseline information of eight pN+ PSCC patients used for subsequent CGP analysis.

Patients	Age	TNM stage	Pathological Stage	LVI	PLNM	ENE
1	44	pT3N3M0	G2	No	No	Yes
2	49	pT1aN3M0	G2	No	Yes	Yes
3	76	pT2N2M0	G2	No	No	No
4	71	pT3N3M0	G2	Yes	No	Yes
5	65	pT1aN3M0	G1	No	No	Yes
6	54	pT2N1M0	G1	No	Uk	No
7	78	pT4N2M0	G2	Yes	No	No
8	57	pT2N2M0	G2	No	Uk	No

Fresh frozen paired samples from eight pN+ PSCC patients were subjected to comprehensive CGP analysis. TNM stage and pathological stage were determined based on the TNM Staging System for Penile Cancer (8th ed., 2017). PSCC, penile squamous cell carcinoma; CGP, comprehensive genomic profiling; LVI, lymphovascular invasion; PLNM, pelvic lymph node; ENE, extranodal extension; Uk, unknown.

cancers MDPI

Table S2. The mRNA expression level of 19 upregulated genes in eight PSCC patients. The mRNA expression level of 19 potential oncogenes of eight pN+ PSCC patients. The values have been standardized. N, normal epithelial tissue; PCA, penile carcinoma; LM, lymph node metastasis; PSCC, penile squamous cell carcinoma.

STK17A	AIM2	BCAT1	IFI44L	EPSTI1	RGS4	IFIT2	MMP9	PLA2G7	SOAT1	GLIPR1	MEST	WISP1	AMIGO2	BCL2A1	COL11A1	LILRB4	NCEH1	RAB20	
9.060	5.440	4.976	3.800	6.710	4.339	4.366	7.426	5.272	4.912	6.160	7.379	5.094	5.438	6.163	4.451	4.806	5.387	5.959	**N1**
8.785	9.580	6.300	6.371	8.697	4.321	6.315	8.927	8.023	6.258	8.031	6.996	6.147	4.549	6.445	3.953	7.956	8.662	7.295	**N2**
7.827	3.740	6.551	3.636	6.898	4.073	5.111	5.459	5.260	5.357	6.840	6.097	4.085	3.571	4.740	3.653	6.021	4.566	6.087	**N3**
7.575	5.114	5.410	6.774	8.233	4.621	5.956	7.591	4.958	5.622	6.727	6.040	4.315	3.946	5.111	5.941	6.386	4.844	5.749	**N4**
8.198	4.312	5.006	5.522	8.187	4.313	5.752	7.632	4.994	4.591	5.776	5.579	3.755	3.547	4.902	4.048	5.168	4.444	5.297	**N5**
8.067	5.203	5.932	3.883	7.502	4.049	5.022	7.601	5.846	5.120	7.188	4.493	3.260	4.396	4.667	3.794	6.367	4.942	5.794	**N6**
8.146	4.103	5.959	5.685	8.255	4.098	6.516	5.432	4.793	5.062	7.026	6.328	3.886	3.602	4.981	4.277	6.571	4.009	6.450	**N7**
8.789	3.610	6.553	3.238	6.667	5.448	4.573	6.169	6.695	5.357	6.985	5.967	3.795	3.491	4.632	4.032	6.310	4.998	6.676	**N8**
9.427	5.021	8.546	4.927	7.321	4.641	4.969	10.210	6.431	5.334	6.537	7.497	5.799	6.177	6.313	4.854	6.067	6.035	7.298	**PCA1**
9.355	10.225	7.001	7.900	9.115	5.600	8.265	10.023	8.867	7.122	8.073	7.616	6.819	5.099	7.625	6.385	9.095	8.465	7.829	**PCA2**
10.282	9.333	8.101	7.678	10.645	6.729	9.185	10.662	6.806	8.152	9.944	8.546	5.560	7.363	7.547	6.083	8.095	7.246	7.486	**PCA3**
9.225	9.990	7.818	8.127	10.672	5.901	7.781	11.130	8.255	7.613	7.759	9.405	6.244	4.385	7.749	5.227	10.144	6.521	8.370	**PCA4**
9.977	9.857	7.285	7.193	10.706	5.443	7.849	9.738	8.012	7.439	9.042	7.848	5.760	6.526	7.263	5.263	7.919	7.203	7.357	**PCA5**
9.871	7.820	7.459	3.814	6.993	5.311	4.736	7.289	5.729	5.377	8.358	4.553	5.563	6.112	5.242	6.207	5.515	5.102	6.112	**PCA6**
9.838	9.417	9.049	6.295	9.458	5.835	7.447	9.215	6.692	6.924	8.323	8.321	5.223	7.301	6.568	8.100	7.053	6.477	7.317	**PCA7**
9.907	7.867	8.449	6.160	9.523	8.389	7.207	12.088	8.768	7.799	8.580	6.361	5.832	5.251	8.149	5.863	9.818	7.703	8.525	**PCA8**
10.186	9.764	7.074	7.592	9.955	4.472	9.132	12.524	9.051	6.981	8.313	6.051	4.356	5.773	8.389	3.465	9.956	9.197	8.338	**LM1**
9.501	7.555	9.398	6.047	8.509	8.340	9.082	10.235	8.204	7.653	7.688	7.106	6.755	6.901	7.113	11.349	8.796	8.786	8.305	**LM2**
9.233	6.811	7.872	6.308	8.836	6.639	8.425	8.045	6.737	6.795	7.893	9.447	5.598	5.740	7.754	4.874	8.455	7.075	8.251	**LM3**
9.466	9.302	9.557	8.538	10.073	6.949	10.109	10.206	7.277	7.799	8.337	8.018	7.072	5.436	6.991	11.121	10.434	7.043	8.307	**LM4**
9.873	9.862	8.426	7.288	10.585	5.193	9.915	10.209	7.639	7.497	7.939	8.377	6.704	5.738	7.985	8.266	7.442	6.574	7.253	**LM5**
10.162	9.291	6.894	4.978	8.594	4.740	7.603	12.018	7.936	6.667	8.703	6.010	4.172	6.120	7.963	3.799	8.847	8.840	8.295	**LM6**
9.492	9.429	8.348	5.359	9.081	7.396	7.432	8.631	6.598	6.966	8.361	8.636	7.316	7.677	6.580	10.116	7.614	6.500	7.304	**LM7**
10.061	8.122	6.942	6.793	9.891	5.310	9.710	9.817	7.760	7.064	7.819	8.387	7.077	5.314	6.512	6.104	7.738	6.320	7.131	**LM8**

Table S3. The mRNA expression level of potential target genes in 39 penile cancer tissues form the GEO database.

STK17A.	AIM2	BCAT1	IFI44L	EPSTI1	RGS4	IFIT2	MMP9	SOAT1	GLIPR1	MEST	WISP1	AMIGO2	BCL2A1	COL11A1	LILRB4	RAB20	
**1.86 ± 0.89**	**4.24 ± 1.89**	**0.95 ± 1.28**	**0.84 ± 2.08**	**1.77 ± 1.46**	**−0.88 ± 0.98**	**0.68 ± 1.69**	**3.94 ± 1.22**	0.09 ± 0.65	0.13 ± 1.19	**1.37 ± 1.39**	**1.20 ± 1.27**	−0.21 ± 1.47	**1.45 ± 1.37**	**1.64 ± 2.48**	−0.17 ± 0.65	**2.01 ± 1.60**	**Mean ± SD**
**<0.001**	**<0.001**	**<0.001**	**0.016**	**<0.001**	**<0.001**	**0.0160**	**<0.001**	0.404	0.494	**<0.001**	**<0.001**	0.374	**<0.001**	**0.001**	0.108	**< 0.001**	***p***
1.81	5.46	1.99	−0.33	0.90	−0.22	−0.20	4.83	−0.18	−0.25	2.40	2.29	1.44	0.39	4.81	0.30	2.12	**1**
0.76	5.15	2.92	3.33	3.09	−2.27	1.79	3.86	0.19	0.22	−0.27	0.28	1.84	1.12	3.50	0.07	1.21	**2**
2.14	5.62	1.04	3.96	4.55	−0.82	3.01	5.44	0.13	1.19	1.71	1.89	0.26	0.88	7.42	−0.04	0.90	**3**
1.40	3.61	−1.05	3.13	0.74	−0.75	0.64	3.50	−0.33	−1.27	2.51	0.56	−0.80	0.37	0.19	−0.14	1.32	**4**
0.58	6.90	0.05	4.58	4.28	−1.26	2.37	4.35	−0.17	−0.76	1.28	1.35	−0.02	0.83	6.53	−0.04	1.68	**5**
0.84	6.92	1.42	3.03	2.95	−0.70	1.03	4.49	−0.33	−0.50	3.11	0.53	−1.17	0.95	2.38	0.11	1.94	**6**
1.42	5.76	2.26	1.91	3.19	−3.05	0.82	4.65	0.90	1.20	4.08	0.72	0.88	0.51	4.56	−0.33	0.32	**7**
2.69	0.52	−0.11	−1.34	1.33	−1.50	−0.89	3.52	0.92	1.14	2.30	−0.32	2.06	0.05	−1.96	−1.67	1.03	**8**
1.40	5.78	1.92	2.22	2.13	−2.08	1.60	4.58	0.35	−0.38	3.42	1.96	−0.72	1.69	5.18	0.26	1.29	**9**
3.09	3.60	1.52	3.24	0.66	−0.56	1.52	3.50	0.44	0.43	3.31	0.10	1.04	0.88	−1.42	−0.36	0.32	**10**
1.93	2.00	−0.04	0.41	−0.22	−0.63	0.35	2.18	0.21	0.51	−0.21	1.38	0.53	−0.16	−0.37	−1.62	1.39	**11**
1.96	2.91	0.80	3.06	2.46	0.48	2.13	3.48	0.88	0.17	3.95	1.74	2.22	0.80	4.71	−1.44	−1.76	**12**
2.11	5.06	1.08	0.25	1.94	−0.73	0.49	4.63	0.30	0.71	1.09	2.51	0.85	1.20	3.25	0.22	0.63	**13**
3.02	6.07	0.87	−1.14	1.02	−0.30	−1.87	4.80	−0.16	1.90	−0.50	2.86	−1.01	3.33	2.12	−0.47	3.17	**14**
2.99	6.46	1.33	2.07	3.96	−1.89	0.85	5.25	1.15	1.40	0.24	1.82	0.93	3.13	1.64	−0.48	3.50	**15**
2.90	5.53	1.03	2.42	2.38	−1.31	3.41	4.85	0.43	2.34	0.96	2.32	−1.33	3.10	0.43	0.52	3.63	**16**
2.32	4.91	0.65	−2.96	0.27	−1.16	−1.21	3.38	−0.98	−0.52	1.65	1.35	−0.95	1.92	2.09	−0.26	3.96	**17**
1.80	5.09	0.97	−0.39	1.73	0.08	0.75	4.72	0.43	1.96	1.69	1.90	−0.71	3.35	3.61	0.79	3.66	**18**
2.42	4.19	2.89	−2.27	1.54	0.02	−1.33	4.58	−0.28	−0.27	0.32	2.58	−1.45	2.66	3.24	0.57	4.04	**19**
0.91	5.07	−0.04	−3.66	−0.16	−2.18	−2.39	4.36	−0.82	−1.12	1.44	1.75	−3.18	2.74	1.23	0.35	3.64	**20**
2.69	4.44	3.01	−3.52	1.24	1.64	−1.40	4.45	0.99	1.28	0.41	4.79	1.55	3.08	4.95	−0.06	2.80	**21**
2.77	4.78	2.28	0.39	2.51	−2.39	2.65	4.34	0.66	0.21	1.63	2.06	−1.77	3.06	−2.37	0.45	3.69	**22**
2.12	5.52	0.61	1.35	3.04	−1.72	3.95	4.63	−0.47	0.86	0.77	1.74	−1.91	2.84	1.42	−0.58	3.82	**23**
2.67	4.98	3.02	2.02	3.25	0.60	3.34	4.19	0.86	0.82	2.24	2.78	0.58	2.93	1.09	0.53	2.98	**24**
1.39	5.16	0.32	−0.80	1.67	−1.55	−0.78	4.23	−0.25	2.17	1.81	0.40	−0.79	2.08	−0.76	0.00	3.64	**25**
1.97	5.93	1.09	1.23	3.67	−1.18	2.17	5.68	0.07	1.15	0.00	1.78	−1.61	3.28	3.20	0.40	3.52	**26**
1.94	5.70	0.94	0.92	2.95	−2.60	1.15	4.94	0.49	−0.51	1.79	0.18	−0.15	1.86	−0.79	0.20	4.20	**27**
1.75	3.97	0.16	0.07	1.91	−1.12	−0.04	2.77	−0.73	0.76	2.91	0.57	−3.00	0.83	0.49	−0.47	4.00	**28**
0.63	2.82	0.29	−0.70	−0.16	−0.61	−0.53	4.25	−0.39	0.82	−1.00	−0.73	0.37	0.00	−1.02	−0.32	0.00	**29**
1.32	3.02	−1.17	2.64	−1.09	−0.18	1.35	3.30	−0.58	−2.14	−0.18	−1.04	−2.97	−1.73	−1.20	−0.97	0.93	**30**
−1.41	−0.91	−0.46	0.00	−0.45	−0.56	0.15	1.08	−1.49	−2.11	−0.77	−0.46	−1.99	3.16	−0.54	0.00	−0.80	**31**

1.81	4.10	0.25	2.61	2.79	−0.45	1.72	5.64	−0.05	−0.33	1.20	−0.79	1.75	−0.31	−0.84	−0.94	−1.42	**32**
1.14	4.81	2.18	2.14	2.65	−0.02	2.29	4.53	0.48	0.58	−0.09	1.91	0.24	1.41	2.50	0.98	1.56	**33**
2.05	5.83	4.32	3.71	3.80	−1.39	2.76	4.39	0.80	−0.88	0.91	0.86	−0.41	1.81	1.62	0.76	2.64	**34**
2.74	1.66	−1.12	−0.86	−0.07	−1.38	−2.37	0.78	−1.50	−1.87	−0.68	−1.21	−1.95	−1.86	−2.06	−0.60	3.32	**35**
1.01	3.17	−0.47	0.40	0.56	0.06	−0.23	0.84	0.07	−2.14	0.01	−0.48	1.98	−0.01	−0.94	−0.89	2.08	**36**
2.35	1.48	−0.89	0.00	1.53	−0.64	0.46	2.24	0.41	−0.81	1.86	1.38	0.71	0.75	1.96	0.00	1.83	**37**
2.34	−0.13	0.03	0.21	0.08	0.43	−0.74	3.63	0.33	−0.66	2.80	1.88	0.27	1.83	1.11	−1.11	0.93	**38**
2.59	2.49	1.32	−0.62	0.44	−0.38	−2.11	2.70	0.66	−0.18	3.24	1.50	0.14	1.81	2.96	−0.39	0.69	**39**

The mRNA expression level of 19 target genes were based on GEO database (Series GSE57955). The mRNA was extracted from 39 fresh frozen penile cancer tissues and 5 corresponding normal tissues submitted to gene expression microarray. The mRNA expression level of NCEH1 and PLA2G7 were undetected in this microarray, other 17 target genes were shown. The values were normalized and calculated by $\log_2$ tumor/control. The one-sample *t* test was used to detect whether the expression differences were significantly. GEO, Gene Expression Omnibus.
